# Stress-Relieving Effects of Japanese Green Tea: Evaluation Using the Molar Ratio of Caffeine and Epigallocatechin Gallate to Theanine and Arginine as an Indicator

**DOI:** 10.3390/foods14010103

**Published:** 2025-01-02

**Authors:** Keiko Unno, Takashi Ikka, Hiroto Yamashita, Yoko Kameoka, Yoriyuki Nakamura

**Affiliations:** 1 Tea Science Center, University of Shizuoka, 52-1 Yada, Suruga-ku, Shizuoka 422-8526, Japan; gp1921@u-shizuoka-ken.ac.jp (Y.K.); yori.naka222@u-shizuoka-ken.ac.jp (Y.N.); 2 Faculty of Agriculture, Shizuoka University, 836 Ohya, Suruga-ku, Shizuoka 422-8529, Japan; ikka.takashi@shizuoka.ac.jp (T.I.); yamashita.hiroto@shizuoka.ac.jp (H.Y.); 3 Institute of Tea Science, Shizuoka University, 836 Ohya, Suruga-ku, Shizuoka 422-8529, Japan; 4 Research Institute of Green Science and Technology, Shizuoka University, 836 Ohya, Suruga-ku, Shizuoka 422-8529, Japan

**Keywords:** theanine, arginine, caffeine, epigallocatechin gallate, green tea, stress reduction

## Abstract

The major components of tea leaves and their infusions were analyzed for various types of green tea available in Japan in 2022. Almost all the green teas used were from the first crop, known for their high amino acid content. The amino acids theanine and arginine in green tea have been shown to reduce stress. On the other hand, epigallocatechin gallate (EGCG) and caffeine, the major components of green tea, counteract the effects of theanine and arginine. We have shown that the CE/TA ratio, which is the ratio of the molar sum of caffeine (C) and EGCG (E) to the molar sum of theanine (T) and arginine (A), can be used to evaluate the stress-relieving effects of each green tea. Green tea with a CE/TA ratio smaller than 3 can be expected to have a stress-reducing effect. The CE/TA ratios of the tea leaves and infusions of Gyokuro, Sencha, and Tamaryokucha were less than 3, indicating that these teas are expected to have stress-relieving effects. In addition, when the same tea leaves were infused repeatedly, it was found that most of the amino acids were eluted by the first and second cups; therefore, no stress-relieving effect could be expected after the third cup.

## 1. Introduction

The data on Japanese green tea chemistry and how to brew Japanese green tea that are now the basis for green tea were established about 50 years ago [1,2,3,4,5]. These data were based on scientific evidence that was sufficiently reliable. However, at that time, green tea was made from traditional cultivars of tea plants, which are different from the tea plants that are widely cultivated today [6], and the processing methods have also since been improved. To clarify the chemical evidence of Japanese green tea that is widely distributed in Japan today, we measured and compared the main components contained in Japanese green tea and investigated changes in the elution components due to differences in the elution method. In addition, we focused on the stress-reducing effects of Japanese green tea and examined what types of green tea and brewing methods could be expected to have stress-reducing effects.

It has recently been reported that the catechin composition and antioxidant activity differ depending on the processing of the tea leaves [7]. In this paper, we focus on the composition ratio of the amino acids (theanine and arginine), catechins, and caffeine in green tea. Theanine and arginine, which are amino acids contained in green tea, have stress-reducing effects, but it has been shown that the main catechin, epigallocatechin gallate (EGCG), and caffeine have an effect that cancels out the effects on stress [8]. Therefore, it is possible to evaluate whether green tea and its infusion can be expected to have a stress-reducing effect by determining the ratio of the molar sum of caffeine (C) and EGCG (E) to the molar sum of theanine (T) and arginine (A) (CE/TA ratio) [9,10]. Previous studies have shown that stress can be reduced if the CE/TA ratio is less than 3, as demonstrated in animal experiments and clinical studies [10,11].

**Figure 1:**
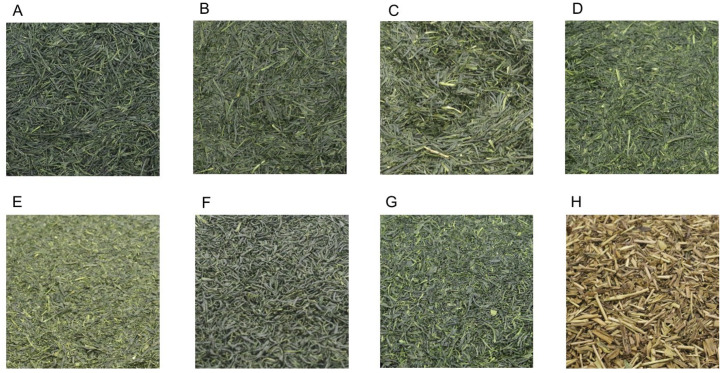
Photos of Japanese green tea. Gyokuro (**A**) is green tea produced from sprouts shaded from direct sunlight. The tea leaves are dark green and needle-shaped. Sencha (**B**–**E**) and Tamaryokucha (**F**,**G**) are produced from tea leaves grown under direct sunlight. Sencha is produced with a needle-like shape. Superior Sencha (**B**) and superior deep-steamed Sencha (**D**) are plucked during the highest quality period. Standard Sencha (**C**) and deep-steamed Sencha (**E**) are harvested at the optimum plucking time when they have advanced in growth to increase yield. Deep-steamed sencha (**D**,**E**) is made by steaming for a longer time than the typical steeping time. Pan-fired Tamaryokucha (**F**) and steamed Tamaryokucha (**G**) have a curled shape. Hojicha (**H**) is Sencha or coarse tea roasted until it turns brown.

The following eight types of Japanese green tea were selected. Gyokuro, superior Sencha, standard Sencha, superior deep-steamed Sencha, deep-steamed Sencha, pan-fired Tamaryokucha, steamed Tamaryokucha, and Hojicha (roasted green tea) (Figure 1). Ninety-four varieties of green tea were purchased from tea-growing regions throughout Japan. Sixty-four of the purchased teas were selected as typical green teas for this test and blended to make test materials for each tea type.

Japanese green tea is usually made from immature new shoots. This is because as they mature, the free amino acids such as theanine decrease [12], and the umami and sweetness also decrease. Theanine, which is synthesized in the roots of tea plants, is transported to the new shoots via the stems [12]. Theanine is quickly metabolized into glutamic acid and ethylamine, and the carbon derived from ethylamine is mainly converted into catechin, an astringent component, but this conversion is inhibited by shading [12]. The intensity of the shading affects the chemical composition of the new shoots [13]. Sencha is green tea grown in direct sunlight and is rich in catechins. Gyokuro is a green tea cultivated by covering the tea field with a reed screen or black cheesecloth for approximately 20 days to shade sunlight as soon as the sprouts appear [14]. Therefore, Gyokuro is a high-grade green tea with a high theanine content (Figure 1A). The shading also gives Gyokuro a unique green-laver-like aroma.

The green teas used in this experiment were from the first crop, except for that of Hojicha. The quality of the first crop of tea is better than that of the second and other teas plucked later. This is because the buds stop growing from the previous fall, allowing the accumulated nutrients to be used for the first crop of tea, and because the growth rate of the shoots is slower than that of the leaves plucked afterwards. Therefore, the green tea used in this experiment belongs to the higher quality ones, i.e., those with high theanine content, in each type of green tea.

Furthermore, there are optimal times to pluck the sprouts [15]. Since the constituents of green tea harvested at different plucking periods vary [16], we are also interested in the effects of differences in harvest time on the stress-relieving effect. Green teas produced when the tea leaves are plucked at their highest quality are superior Sencha (Figure 1B) and superior deep-steamed Sencha (Figure 1D). On the other hand, as maturity progresses, yields increase and profitability improves. The green teas produced from plucking during these periods are standard Sencha (Figure 1C) and deep-steamed Sencha (Figure 1E).

In the tea manufacturing process, Japanese green tea is first steamed or roasted to deactivate oxidative enzymes in the tea leaves [17]. Green tea made with standard steaming time is called Sencha (Figure 1B,C), whereas green tea made by steaming longer than the standard steaming time (about 60 to 120 s) is called deep-steamed Sencha (Figure 1D,E). The steamed leaves are then dried with hot air and pressure while being stirred and further dried while being shaped to form the tea. Steaming time affects the color of green tea [18]. Longer steeping reduces astringency and increases sweetness but weakens the aroma. Also, because of the long steeping process, the leaves tend to become finer and powdery during production [19]. This increases the apparent density and darkens the color of the infusion.

Another method is to inactivate oxidative enzymes in tea leaves by roasting the plucked tea leaves in a tea leaf parcher [17]. After that, they are dried under heat and pressure while being stirred. In this case, the tea leaves become round and curved due to friction with each other. The greenish smell disappears and a distinctive aroma develops. Green tea produced using this Chinese method is pan-fired Tamaryokucha (Figure 1F). After steaming to stop the action of oxidative enzymes, tea leaves are heated and dried in a tea leaf parcher under stirring to produce the steamed Tamaryokucha (Figure 1G). Hojicha (roasted green tea, Figure 1H) is made by roasting Sencha or coarse tea over high heat to give it a roasted aroma and has a golden-brown appearance. The Sencha and coarse tea used to make Hojicha is not the first crop of tea.

## 2. Materials and Methods

### 2.1. Selection of Japanese Green Tea

The following eight types of Japanese green tea were purchased from tea-growing regions throughout Japan: Gyokuro, superior Sencha, standard Sencha, superior deep-steamed Sencha, deep-steamed Sencha, pan-fired Tamaryokucha, steamed Tamaryo-kucha, and Hojicha.

Nine Gyokuro teas were purchased from Fukuoka Prefecture (Yame), Kyoto Prefecture (Uji), and Shizuoka Prefecture (Asahina), from which six were selected. Eighteen pieces each of superior Sencha and standard Sencha were purchased from the following prefectures: Niigata, Ibaraki, Saitama, Kanagawa, Shizuoka, Kyoto, Miyazaki, and Kagoshima. Nine items were selected from among them. Ten pieces each of superior deep-steamed Sencha and deep-steamed Sencha were purchased from the following prefectures: Shizuoka, Mie, Kagoshima, and Saitama. Nine were selected from among them. Eight pan-fired Tamaryokucha were purchased from Miyazaki and Kagoshima prefectures and used for the test. Eight steamed Tamaryokucha teas were purchased from Nagasaki and Saga prefectures and used for the test. Twelve Hojicha teas were purchased from Saitama, Shizuoka, Shiga, and Kagoshima prefectures. Six were selected from among them.

For each type of Japanese green tea, some typical green teas were selected. These teas were blended for test material.

### 2.2. Infusion of Japanese Green Tea

Three groups independently conducted infused tests on each Japanese green tea. Hot water was boiled and set at a predetermined temperature. To maintain the temperature, a fixed amount of hot water was placed in a warming pot. The hot water was quickly added to a teapot (Kyusu) containing the determined amount of tea leaves, and the tea was eluted at the prescribed infusion time. In the test using cold water at 5 °C, the sample was allowed to stand for a defined time without shaking. Three samples were obtained for each Japanese green tea test point. The resulting infusion solutions were submitted for analysis.

### 2.3. Measurement of Free Amino Acids

The concentrations of amino acids in tea leaves and tea-infused solutions were determined through high-performance liquid chromatography (HPLC). The free amino acid content was determined for nine amino acids under the following measurement conditions, with modifications of the methods outlined by Goto et al. [20] and Yamashita et al. [21].

Measures of 10 mg of polyvinylpolypyrroridone and 5 mL of ultrapure water were added to 10 mg of dried green tea powder sample. Shaking extraction (130 strokes/min) was performed for 1 h at room temperature. The extract was centrifuged (2000× *g*, 30 min). The obtained supernatant was filtered through a 0.45 μm cellulose acetate filter (Advantec, Tokyo, Japan). The filtrate was stored at −30 °C until analysis using HPLC.

The tea infusion was filtered through a 0.45 μm cellulose acetate filter (Advantec, Tokyo, Japan) and diluted with ultrapure water. The filtrate for analysis was subjected to HPLC using the o-phthalaldehyde (OPA) precolumn derivatization system after adding homoserine as an internal standard.

The HPLC system (Shimadzu, Kyoto, Japan) used for the analysis consisted of two pumps (LC-10AT), a degasser (DGU-20A5R), a column oven (CTO-10Avp), a fluorescence detector (RF-20A), a system controller (SCL-10Avp), and an autosampler (SIL-10AF).

The HPLC analysis conditions were set as follows: injection volume, 5 μL; separation column (75 mm × 4.6 mm × 5 μm, Ascentis Express C18 column, Shigma-Aldrich Co. LLC, St. Louis, MI, USA); column oven temperature, 40 °C; excitation wavelength, 340 nm; fluorescence wavelength, 450 nm.

Analysis was performed using mobile phase A (5% (*v*/*v*) acetonitrile, 5 mM citrate buffer (pH 6.0) and mobile phase B (70% (*v*/*v*) acetonitrile, 5 mM citrate buffer (pH 6.0)) at a flow rate of 1.0 mL/min under the gradient conditions shown in Table 1.

The analysis was performed for nine amino acids (L-aspartic acid, L-asparagine, L-glutamine, L-glutamic acid, L-serine, L-arginine, L-alanine, L-theanine, and γ-aminobutyric acid) using an internal standard method. The reagents used in chromatography were purchased from Merck (Darmstadt, Germany) and FUJIFILM Wako Pure Chemical Corporation (Osaka, Japan). The standards used in chromatography were purchased from FUJIFILM Wako Pure Chemicals Corporation (Osaka, Japan).

### 2.4. Measurement of Catechins and Caffeine

A measure of 5 mL of 50% (*v*/*v*) acetonitrile was added to 25 mg of dry powdered tea leaves and mixed well. The mixture was shaken for 1 h at room temperature (130 strokes/min). The supernatant obtained using centrifugation (2000× *g*, 30 min) was filtered through a 0.45 μm polytetrafluoroethylene (PTFE) filter (Advantec, Tokyo, Japan). The resulting filtrate was stored at −80 °C until analysis using HPLC.

The green tea infusion was filtered through a 0.45 μm PTFE filter (Advantec, Tokyo, Japan), diluted with 50% (*v*/*v*) acetonitrile, and subjected to analysis using HPLC under the following measurement conditions, with modifications of the method outlined by Yamashita et al. [21].

The HPLC system (Shimadzu, Kyoto, Japan) used for the analysis consisted of the following: pumps (2 sets, LC-10ADvp), a degasser (D6U-14A), a column oven (CPO-20AC), a photodiode array detector (SPD-M20A), a system controller (SCL-10Avp), and an autosampler (SLC-10ADvp).

The HPLC analysis conditions were set as follows: extraction volume, 5 μL; separation column (75 mm × 4.6 mm × 2.6 μm, SunShell C18 column, ChromaNik Technologies Inc., Osaka, Japan); column oven temperature, 40 °C; photodiode array measurement wavelength range, 199–400 nm.

Mobile phase A consisted of 1909:90:1 (*v*/*v*/*v*) mL of ultrapure water/acetonitrile/85% (*v*/*v*) phosphoric acid and mobile phase B consisted of 999:1000:1 (*v*/*v*/*v*) mL of ultrapure water/acetonitrile/85% (*v*/*v*) phosphoric acid at a flow rate of 1.0 mL/min. The gradient conditions are shown in Table 1.

Seven catechins ((+)-gallocatechin (GC), (+)-catechin ((+) C), (−)-epicatechin (EC), (−)-epigallocatechin (EGC), (−)-catechin gallate (CG), (−)-epicatechin gallate (ECG), (−)-epigallocatechin gallate (EGCG)) and caffeine were determined through the area percentage method. Reagents used in chromatography were purchased from Merck (Darmstadt, Germany) and FUJIFILM Wako Pure Chemical Corporation (Osaka, Japan). Standards used in chromatography were purchased from FUJIFILM Wako Pure Chemicals Corporation (Osaka, Japan).

### 2.5. Statistical Analysis

The data are presented as the mean ± standard error. Statistical analysis was performed using one-way analysis of variance, and statistical significance was set at *p* < 0.05. Confidence intervals and the significance of differences in means were estimated using the Tukey–Kramer significant difference method.

## 3. Results

### 3.1. Tea Components in Each Green Tea Leaf

The main constituents of the tea leaves were analyzed for the various tea leaves, which were blended to make test materials for each tea type. The average of three measurements of the content (mg) of each component per gram of tea leaves is shown in Table 2. For theanine, arginine, caffeine, and EGCG, values converted to moles (μmol) are shown in red. Gyokuro contained more amino acids and caffeine than any other green tea, while catechins were the lowest in these teas, except for Hojicha. Steamed Tamaryokucha had the next highest levels of theanine and glutamine after Gyokuro, and the lowest levels of catechins, with the exception of Hojicha and Gyokuro. Superior Sencha had higher amino acid, caffeine, and EGCG contents than standard Sencha. Superior deep-steamed Sencha had higher amounts of theanine and arginine than deep-steamed Sencha. Pan-fired Tamaryokucha had the lowest theanine content other than Hojicha, while caffeine and EGCG were the second highest after superior Sencha. Amino acids such as theanine were broken down in Hojicha through heat treatment. The amounts of EGCG, EGC, ECG, and EC among catechins were also significantly reduced in Hojicha compared to in other green teas. On the other hand, caffeine sublimated upon heating but was not decomposed. The amount of caffeine in the Sencha used as fresh material was reflected in this value.

Based on the results, the CE/TA ratio, which is the ratio of the molar sum of caffeine and EGCG to the molar sum of theanine and arginine, was determined. Previous studies have shown that if this value is smaller than 3, a stress-reducing effect can be expected [9,10]. Therefore, the results suggest that Gyokuro, steamed Tamaryokucha, superior Sencha, standard Sencha, superior deep-steamed Sencha, deep-steamed Sencha, and pan-fired Tamaryokucha are expected to have stress-reducing effects.

**Figure 2:**
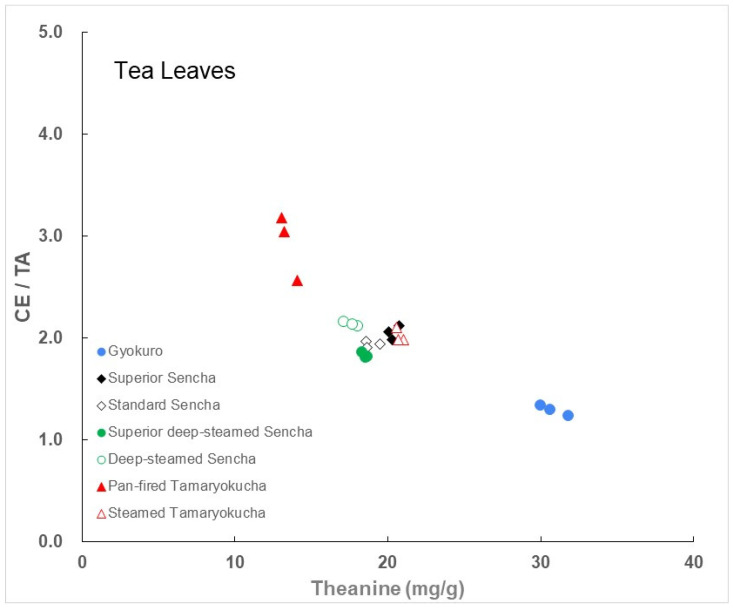
The relationship between the theanine content and CE/TA ratio in each green tea. The results of the three measurements for each green tea are shown here. Values for Hojicha are not included.

The relationship between the CE/TA ratio and theanine content for each green tea is shown in Figure 2. The results of the three measurements for each green tea are shown here. Since the CE/TA ratio of Hojicha was significantly higher than those of other green teas because amino acids such as theanine were significantly reduced through heat treatment, it was not included in this figure.

A high theanine content is an indicator of Japanese green tea quality. From this perspective, Gyokuro, steamed Tamaryokucha, superior Sencha, standard Sencha, superior deep-steamed Sencha, deep-steamed Sencha, and pan-fired Tamaryokucha contained the most theanine, in that order. The leaves of superior Sencha, standard Sencha, superior deep-steamed Sencha, deep-steamed Sencha, and steamed Tamagawa had very similar theanine contents and CE/TA ratios. The CE/TA ratio of pan-fired Tamaryokucha was also smaller than 3.

### 3.2. Tea Components in the Infused Solution of Each Green Tea

Typical values for the infusion of each Japanese green tea are shown in Table 3. In the case of Gyokuro, 60 mL of hot water at 60 °C was poured over 10 g of tea leaves, and the results for infusion after 2 min are shown. For superior Sencha, 170 mL of hot water at 70 °C was poured into 6 g of tea leaves, and the infusion was obtained after 1 min. For standard Sencha and deep-steamed Sencha, pan-fired Tamaryokucha, and steamed Tamaryokucha, 260 mL of hot water at 90 °C was poured into 6 g of tea leaves, and the infusion was obtained after 1 min. For Hojicha, 390 mL of hot water at 90 °C was poured into 9 g of tea leaves, and the infusion was obtained after 1 min.

The elution rate of the constituents in tea leaves varies depending on the amount of tea leaves, hot water temperature, hot water volume, and infusion time used for infusing. Table 3 shows examples of the concentration of each component of Japanese green tea and the CE/TA ratio of tea eluate. In general, Gyokuro tea leaves should be infused at a lower temperature (50–60 °C) and a longer infusion time (2 to 2.5 min) to allow sufficient elution of amino acids and suppress the elution of catechins and caffeine. Because Gyokuro used a larger amount of tea leaves, a smaller amount of hot water used for infusion, and a longer infusion time than superior Sencha, Gyokuro extracted 10 times more theanine and 7 times more caffeine than superior Sencha. Therefore, Gyokuro had a lower CE/TA ratio than that of superior Sencha (Table 3). Although the amounts of amino acids in the tea leaves of deep-steamed Sencha were lower than those of standard Sencha (Table 2), the amounts of amino acid eluted from deep-steamed Sencha were higher than those of standard Sencha (Table 3). This is thought to be mainly due to the finer tea leaves resulting from the longer steaming time [19]. Pan-fired Tamaryokucha had lower amounts of amino acids in the tea leaves, but the caffeine and catechins were similar to those of standard Sencha (Table 2). However, caffeine and catechins in the infusion of pan-fired Tamaryokucha were lower than those of standard Sencha (Table 3). In addition, the amount of each component in the steamed Tamaryokucha tea leaves was close to that of superior Sencha (Table 2). The infusion of steamed Tamaryokucha shown in Table 3 was infused at 90 °C for 1 min, but when compared to the infusion of the tea for 1 min at 70 °C, the amounts of eluted amino acids, caffeine, and catechins were lower in steamed Tamaryokucha than in superior Sencha. Since Tamaryokucha is not subjected to as much rolling pressure during the tea making process as Sencha, it is thought that the elution rate is lower [22].

**Figure 3:**
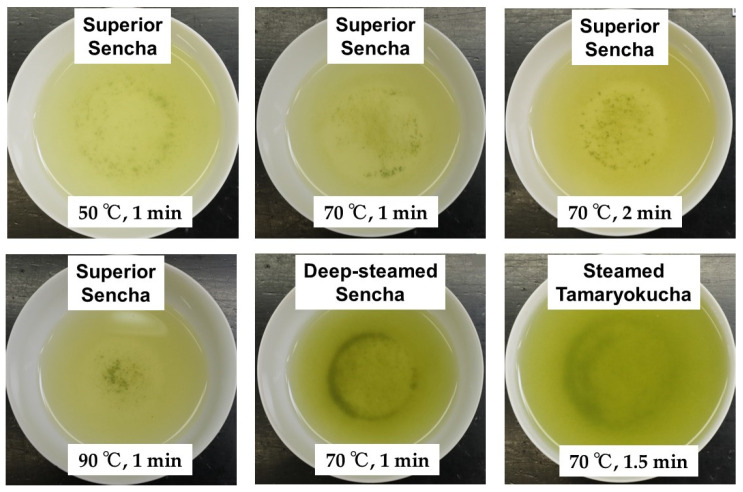
Infused solution of Japanese green tea. Six grams each of superior Sencha, deep-steamed Sencha, and steamed Tamaryokucha were infused with 170 mL and 260 mL of hot water at 50, 70, and 90 °C for 1 to 2 min.

As shown in Figure 3 using superior Sencha as an example, the higher the temperature of the infused water and the longer the elution time, the darker the color of the green tea infusion was. Deep-steamed Sencha and steamed Tamaryokucha were darker in color than superior Sencha under the same infusion conditions (Figure 3). The results showed that the CE/TA ratio was less than 3 under all green teas except Hojicha. The CE/TA ratio of tea infusion of Hojicha was significantly higher because the amounts of amino acids and EGCG in the tea leaves decreased while the amount of caffeine remained the same as that of other teas.

**Figure 4:**
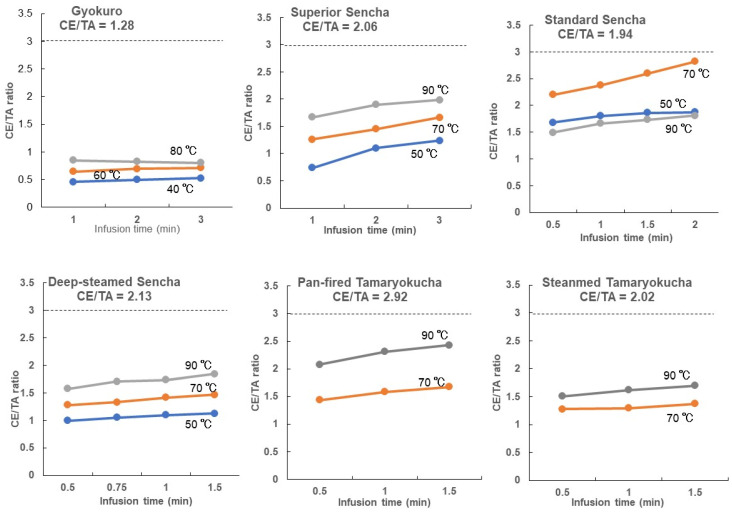
Changes in CE/TA ratio of each Japanese green tea when the amount of tea leaves and hot water was kept constant and the temperature of hot water and extraction time were varied. All of the values were lower than the CE/TA ratio of 3 (broken line).

Next, we examined whether the CE/TA ratio changed with water temperature and infusion time. Figure 4 shows the change in CE/TA ratio for each Japanese green tea when the amounts of tea leaves and hot water are kept constant and the temperature of the hot water and infused time are varied. The CE/TA ratio was higher for each green tea at higher water temperatures and longer infusion times (Figure 4).

**Figure 5:**
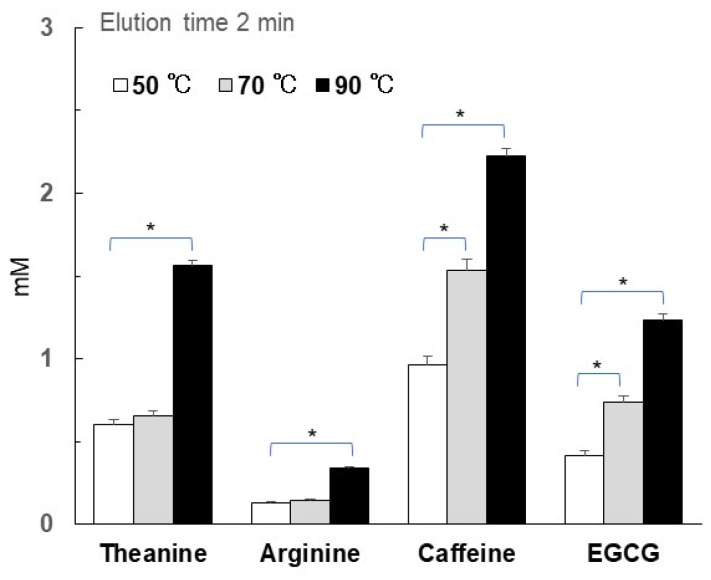
Effect of water temperature on the elution of theanine, arginine, caffeine, and EGCG in standard Sencha. A measure of 6 g of standard Sencha (CE/TA = 1.94) was infused with 260 mL of hot water at 90 °C for 2 min. Each column represents the mean ± SEM (*n* = 3). (* *p* < 0.05, Tukey–Kramer significant difference method).

In the case of standard Sencha, the CE/TA ratio was almost the same when infused at 50 °C and 90 °C (Figure 4). However, theanine and arginine increased 2.6-fold, caffeine 2.3-fold, and EGCG 3-fold at 90 °C compared to at 50 °C (Figure 5). Thus, even though the CE/TA ratio was the same at 50 °C and 90 °C, there was a difference in the amounts of eluted components. In addition, the elution of amino acids did not increase much at 70 °C compared to at 50 °C (Figure 5), but their elution amounts could change compared to in previous reports [23,24]. Since CE/TA ratios are strongly influenced by the amount of theanine, the 70 °C results were higher than those at 50 and 90 °C. However, the CE/TA ratios for standard Sencha at 70 °C with infusion times of 0.5 to 2 min were all less than 3.

**Figure 6:**
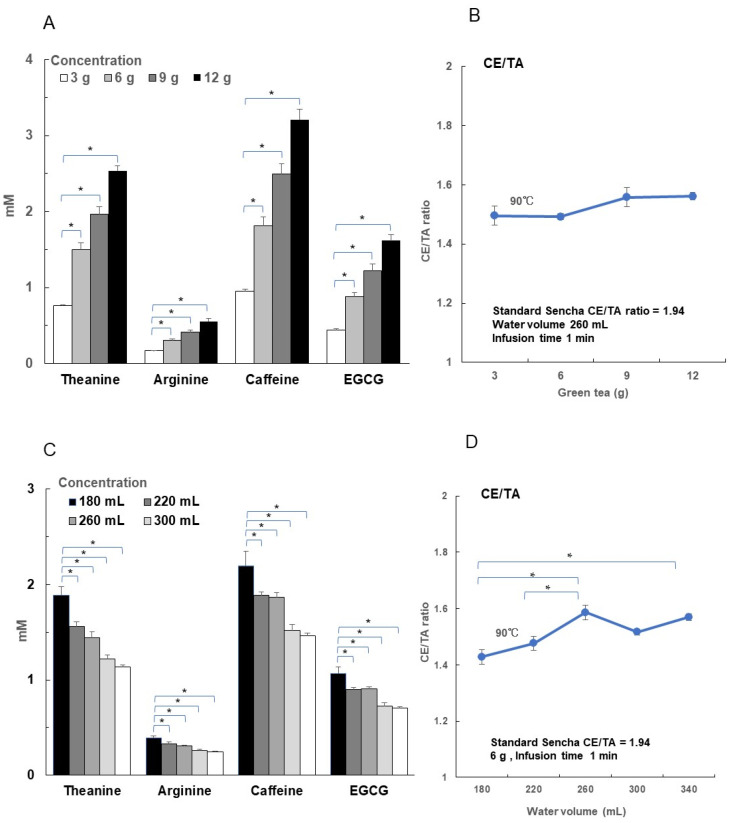
Effects of the amount of tea leaves and the infusion water volume on CE/TA ratio. A measure of 3–12 g of standard Sencha (CE/TA = 1.94) was infused with 260 mL of hot water at 90 °C for 1 min (**A**,**B**). The relationship between the concentrations of theanine, arginine, caffeine, and EGCG in the resulting infusion and the amount of tea leaves is shown in A. The relationship between the tea leaf volume and CE/TA ratio is shown in B. A measure of 6 g of standard Sencha (CE/TA = 1.94) was infused with 180–340 mL of hot water at 90 °C for 1 min (**C**,**D**). The relationship between the concentrations of theanine, arginine, caffeine, and EGCG in the resulting infusion and tea brewing water infusion volume is shown in (**C**). The relationship between the infusion water volume and CE/TA ratio is shown on (**D**). Each column and point represent the mean ± SEM (*n* = 3) (* *p* < 0.05, Tukey–Kramer significant difference method).

Furthermore, we investigated whether the CE/TA ratio changed with the amount of tea leaves used and the amount of hot water used for infusion. When 3–12 g of standard Sencha tea (CE/TA ratio 1.94) was infused with 260 mL of hot water at 90 °C for 1 min, theanine, arginine, caffeine, and EGCG increased in elution amount in proportion to the amount of tea leaves used (Figure 6A). The CE/TA ratio increased with the amount of tea leaves, but the change was not significant (Figure 6B).

On the other hand, the volume of hot water used to infuse 6 g of standard Sencha tea for 1 min was increased from 180 mL to 340 mL, and the concentrations of all green tea components in the infusion decreased (Figure 6C). However, theanine and arginine decreased to about 60% at 340 mL compared to at 180 mL, while caffeine and EGCG decreased to about 70%. Thus, it was found that the CE/TA ratio of tea eluate was significantly higher with increasing infusion water volume (Figure 6D). Comparing these data in terms of the CE/TA ratio shows that even when the same tea leaves are used, differences in hot water volume relative to the amount of tea leaves used have different effects on the elution efficiency of each component. In other words, the higher the hot water volume, the more caffeine and EGCG are eluted relatively.

**Figure 7:**
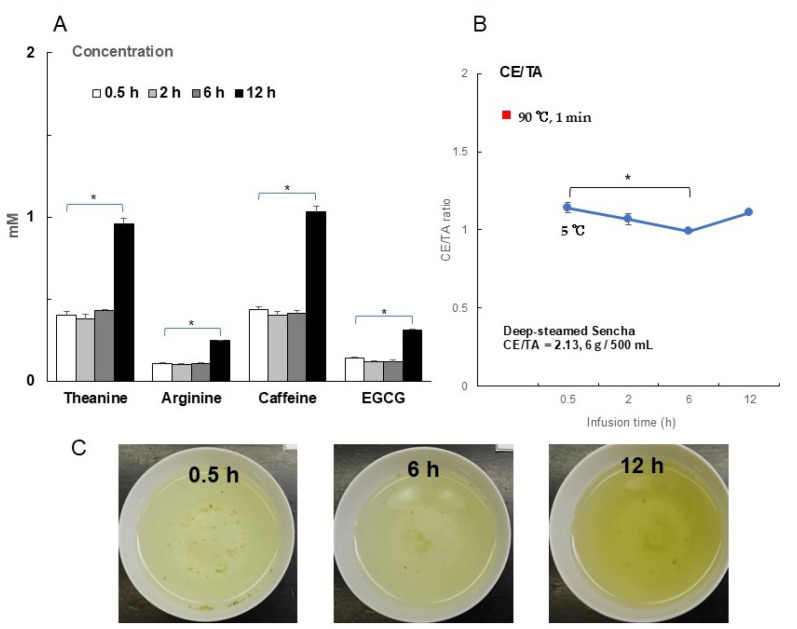
Effect of the cold-water brewing time on the CE/TA ratio of infusion. A measure of 6 g of deep-steamed Sencha (CE/TA = 2.13) was infused with 500 mL of cold water at 5 °C for 0.5–12 h. The relationship between the concentrations of theanine, arginine, caffeine, and EGCG and time (**A**). The relationship between the infusion time and CE/TA ratio at 5 °C. For comparison, the CE/TA ratio at 90 °C for 1 min was added (**B**). Photos of infused solution for 0.5, 6, and 12 h (**C**). Each column and point represent the mean ± SEM (*n* = 3) (* *p* < 0.05, Tukey–Kramer significant difference method).

Next, cold-water brewing was examined: 6 g of deep-steamed Sencha (CE/TA = 2.13) was infused in 500 mL of cold water (5 °C). Due to the low penetration of water into the dried tea leaves in cold water [25], there was little change in the infusion concentrations of theanine, arginine, caffeine, and EGCG from 0.5 to 6 h, but after 12 h, the concentrations increased to more than double those at 0.5 h (Figure 7A). In fact, the increase in concentration can be seen from the color of the infused tea (Figure 7C). The CE/TA ratio of deep-steamed Sencha when brewed at 90 °C was 1.73 (Table 3), but it decreased to 1.1 at 5 °C (Figure 7B). This means that at 5 °C, there is less elution of caffeine and EGCG compared to that of theanine and arginine.

### 3.3. Change in CE/TA Ratio with Number of Infused Cups

**Figure 8:**
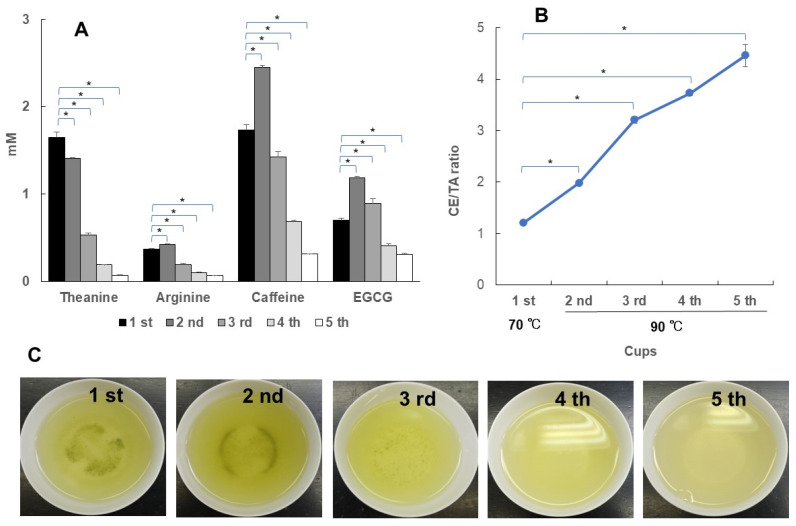
Changes in the amounts of elution constituents and the CE/TA ratio from the first to the fifth cup. A measure of 6 g of superior Sencha (CE/TA = 2.06) was infused with 170 mL of hot water at 70 °C for 1 min (first cup). Next, it was infused with 170 mL of hot water at 90 °C for 20 s (second cup). The third, fourth, and fifth cups were brewed in the same manner. The relationship between the concentrations of theanine, arginine, caffeine, and EGCG and cups (**A**). The relationship between cups and CE/TA ratio (**B**). Photos of infused solution for the first to fifth cups (**C**). Each column and point represent the mean ± SEM (*n* = 3) (* *p* < 0.05, Tukey–Kramer significant difference method).

**Figure 9:**
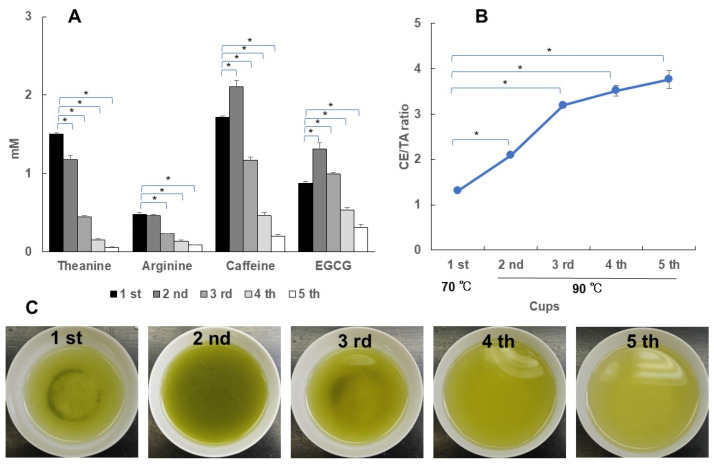
Changes in the amounts of eluting constituents and the CE/TA ratio from the first to the fifth cup. A measure of 6 g of superior deep-steamed Sencha (CE/TA = 1.82) was infused with 170 mL of hot water at 70 °C for 1 min (first cup). Next, it was infused with 170 mL of hot water at 90 °C for 20 s (second cup). The third, fourth, and fifth cups were brewed in the same manner. The relationship between the concentrations of theanine, arginine, caffeine, and EGCG and cups (**A**). The relationship between cups and CE/TA ratio (**B**). Photos of infused solution for the first to fifth cups (**C**). Each column and point represent the mean ± SEM (*n* = 3) (* *p* < 0.05, Tukey–Kramer significant difference method).

Usually, multiple cups of Japanese green tea are brewed from the same leaves. Therefore, using superior Sencha (CE/TA = 2.06), the first cup was infused with hot water at 70 °C for 1 min, and the second and subsequent cups were infused with hot water at 90 °C for 20 s. The concentration of theanine in the elution solution decreased as the number of infused times increased (Figure 8A). On the other hand, caffeine and EGCG were highest in the second cup brewed at 90 °C and decreased thereafter. As a result, the CE/TA ratio increased as the number of infused cups increased (Figure 8B). After the third cup, the theanine elution amount decreased significantly, resulting in a CE/TA ratio of more than 3. This suggests that the stress-relieving effect decreases after the third cup. Photographs from the first to the fifth cups of the actual infused solution are shown in Figure 8C; the color became darker in the second cup compared to in the first cup and lighter from the third cup onward. Superior deep-steamed Sencha (CE/TA = 1.82) was also examined in the same way (Figure 9). Again, the CE/TA ratio was greater than 3 after the third cup. Compared to the case of superior Sencha, the superior deep-steamed Sencha showed a darker green color even in the fourth and fifth cups (Figure 9C), but the CE/TA ratio was above 3. It was clear that theanine and arginine, which show stress-relieving effects in Japanese green tea, were mostly eluted by the first and second cups.

## 4. Discussion

Animal studies on the stress-relieving effects of tea leaves with different CE/TA ratios revealed that the CE/TA ratio should be less than 3 [11]. In addition, the results of animal experiments and clinical trials suggest that the amount of theanine required for humans to obtain stress-relieving effects is at least approximately 100 mg/day [11,26]. Seven of the Japanese green teas used in this study, with the exception of Hojicha, had CE/TA ratios of 3 or less (Table 2). The CE/TA ratios of all seven types of tea extracts were less than 3 (Table 3, Figure 4). These seven Japanese tea infusions investigated in this study are expected to have stress-relieving effects. The fact that no tea had a CE/TA ratio of 3 or higher at the tea leaf level indicates that all the Japanese green teas used in this study were high-quality green teas with high contents of amino acids such as theanine and arginine. Although not all Japanese green teas have a CE/TA ratio of 3 or less, since all Japanese green teas used in this study were from the first crop, it may be safe to assume that any first crop of Japanese green tea has a CE/TA ratio of 3 or less.

Comparing the content of components in the tea leaves obtained in 2022 with previously reported values, it was found that the studied green teas had higher amino acid contents and lower catechin contents [3,24,27,28,29]. This may be due to the increased use of fertilizers compared to previously [30], differences in cultivars [6], and an increase in the amount of tea grown under cover [31].

When Gyokuro is brewed at low temperatures (40–60 °C) for at least two minutes, it produces a rich flavor with a good balance of umami and astringency [32,33]. Gyokuro is usually drank in small 30–50 mL tea cups, with about 1–2 cups typically consumed per day [5]. In fact, in the case of Gyokuro, it has been shown that one or two small cups of tea are sufficient to obtain its stress-relieving effect. Gyokuro is rich in theanine but also contains a lot of caffeine. Although there have been no reports of theanine being harmful [34,35], the generally accepted amount of caffeine that an adult can consume in a day is 400 mg [36], so drinking more than 177 mL of Gyokuro every day could result in an excessive caffeine intake.

The more commonly consumed Sencha is brewed with hot water at 90 °C and steeped for about one minute, while superior Sencha is often brewed at a slightly lower temperature of 70 °C. Several cups of Sencha are typically consumed per day. For superior Sencha, standard Sencha, deep-steamed Sencha, and steamed Tamaryokucha, 400–500 mL is sufficient for stress relief, and drinking several cups of these green teas per day is expected to have a stress-relieving effect. The pan-fired Tamaryokucha used in this study contained less theanine than other Japanese green teas (Table 3), suggesting that 700 mL or more was necessary to achieve stress-relieving effects.

Multiple cups of Japanese green tea are generally consumed from one brewing of the same tea leaves. However, caution should be taken when trying to obtain a stress-relieving effect by drinking several cups of green tea; the CE/TA ratio was found to be more than 3 after the third cup. Theanine and arginine, components showing stress-relieving effects, were found to be mostly eluted after the first and second cups. The color of the extract of the superior deep-steamed Sencha was dark green even after the fourth to fifth cup, but the CE/TA ratio was above 3 (Figure 9). This indicates that the presence or absence of stress-relieving effects cannot be inferred from the color of the eluting solution. These results suggest that tea leaves should be replaced appropriately if stress-relieving effects are to be expected.

In recent years, interest in the functionality and safety of green tea infused with cold water has increased [37,38]. Caffeine and EGCG are efficiently eluted at high temperatures but have also been reported to be eluted over time in cold water [24,39]. When tea leaves were extracted in cold water at 5 °C, theanine and arginine elution was greatly reduced, while the elution of caffeine and EGCG was even less than that of theanine and arginine. Thus, a deep-steamed Sencha with a CE/TA ratio of 1.73 after 1 min of infusion at 90 °C was significantly reduced from 1 to 1.1 at 5 °C. Although the CE/TA ratio was sufficiently low, 600 mL of green tea after 12 h of infusion time would be required to provide the amount of theanine needed for stress relief. It should be noted that even tea with a high CE/TA ratio can be infused with a low CE/TA ratio through cold water extraction, but the amount of theanine in the tea leaves is low.

Hojicha is characterized by its fragrant aroma. In Hojicha, the CE/TA ratio was very high because amino acids and catechins are decomposed by heat, while caffeine is slightly reduced by sublimation but not decomposed. Therefore, in the case of Hojicha, the CE/TA ratio does not seem to be suitable indicator for evaluating its stress-relieving effect.

With regard to the functional properties of green tea, effects on obesity [40], revamping the gut and skin from stress [41], a neuroprotective effect [42], and effects on cognition and mood [43] have been reported. In addition, it has been previously reported that the physicochemical properties of green tea vary depending on the extraction method [44] and that the content of catechins and caffeine varies depending on the hardness of the water [45]. Brewing time and temperature have also been reported to affect the antioxidant activity of white tea [46]. Further research is needed to determine the kind of tea and how it is brewed and what functions it is expected to have.

Many Japanese people say that drinking green tea calms them down. It is possible that the habit of drinking green tea in Japan may reduce stress without many Japanese people being aware of it. This study is considered scientific proof that this feeling is in fact true.

## 5. Conclusions

Since Japanese green teas recently produced and sold in Japan differ from those reported in the past in terms of cultivars and production methods, we clarified the main components of tea leaves and their infusions in eight types of Japanese green teas sold in Japan in 2022. Based on these results, we inferred that the type of Japanese green tea and how it is brewed can be expected to have stress-relieving effects. All of the Japanese green teas used in this study, with the exception of Hojicha, were from the first crop (i.e., first-grade green teas); thus, seven types of Japanese green tea leaves and their infusions could be expected to have stress-relieving effects. Many Japanese people say that drinking green tea makes them feel relieved. This study is considered scientific proof that this feeling is in fact true.

## Figures and Tables

**Table 1:** HPLC gradient conditions for tea component analyses.

Free Amino Acid Measurement		Catechin and Caffeine Measurements	
Time	Eluent B Concentration (%)	Time	Eluent B Concentration (%)
0.1	5	0.0	10
1.6	12	2.5	10
6.0	22	4.0	30
7.0	95	5.0	30
10.0	95	7.5	80
10.5	5	10.0	80
12.0	0	11.0	10
13.0	0	15.0	10

**Table 2:** Tea components in tea leaves.

mg/g (μmol)	Gyokuro	Superior Sencha	Standard Sencha	Superior Deep-Steamed Sencha	Deep-Steamed Sencha	Pan-Fired Tamaryokucha	Steamed Tamaryokucha	Hojicha
Theanine	30.84	20.37	18.91	18.55	17.65	13.47	20.77	0.32
	177	117	109	106	101	77	119	2
Arginine	9.72	5.63	4.72	6.73	4.90	2.56	4.13	0.30
	56	32	27	39	28	15	24	2
Glutamine	4.96	4.48	3.66	4.35	3.79	1.36	5.07	0.03
Aspartic acid	5.74	2.37	1.79	1.83	1.90	1.58	2.54	0.19
Glutamic acid	4.41	3.00	2.63	2.74	2.51	2.18	2.87	0.07
Asparagine	2.37	0.33	0.16	0.17	0.22	0.14	0.42	0.01
Serine	0.97	0.83	0.70	0.73	0.55	0.47	0.53	0.04
Alanine	0.42	0.23	0.18	0.19	0.19	0.17	0.23	0.03
GABA	0.14	0.18	0.10	0.15	0.15	0.16	0.09	0.01
Caffeine	35.41	31.01	24.70	25.32	26.70	24.87	30.06	20.71
	182	160	127	130	137	128	155	107
EGCG	53.31	67.45	62.16	61.61	63.51	64.29	61.61	9.00
	116	147	136	134	139	140	134	20
EGC	7.65	20.09	25.87	25.92	25.65	31.05	14.80	3.45
ECG	10.97	15.86	13.78	13.95	13.71	13.46	13.11	2.94
EC	3.33	6.33	7.81	7.81	7.30	8.18	5.01	1.22
(+) C	1.49	2.06	1.99	2.06	1.83	1.65	1.47	2.45
GC	0.54	1.37	1.67	2.25	2.29	1.83	1.26	5.74
CG	0.66	1.37	1.17	1.30	1.23	1.13	1.05	1.65
CE/TA ratio	1.28	2.06	1.94	1.82	2.13	2.92	2.02	35.71

GABA, γ-aminobutyric acid; EGC, epigallocatechin; ECG, epicatechin gallate; EC, epicatechin; (+) C, catechin; GC, gallocatechin; CG, catechin gallate. Values of theanine, arginine, caffeine, and EGCG converted to moles (μmol) and CE/TA ratio are shown in red.

**Table 3:** Amount of each component in green tea infusion and the CE/TA ratio of tea eluate.

Green Tea	Gyokuro	Superior Sencha	Standard Sencha	Deep-Steamed Sencha	Pan-Fired Tamaryokucha	Steamed Tamaryokucha	Hojicha
Leaf (g)	10	6	6	6	6	6	9
Water temperature (°C)	60	70	90	90	90	90	90
Water volume (mL)	60	170	260	260	260	260	390
Infusion time (min)	2	1	1	1	1	1	1
Theanine (mg/L)	2806.81	256.14	218.41	240.30	138.13	267.07	3.02
(mM)	16.11	1.47	1.25	1.38	0.79	1.53	0.02
Arginine	721.66	54.16	47.26	57.33	24.04	43.40	2.24
(mM)	4.14	0.31	0.27	0.33	0.14	0.25	0.01
Glutamine	483.36	56.18	45.34	51.12	16.64	63.02	0.23
Aspartic acid	694.30	41.33	26.59	31.16	21.91	44.09	3.01
Glutamic acid	475.04	47.13	36.03	41.12	28.49	45.56	0.80
Asparagine	272.24	5.25	2.24	3.76	1.55	7.03	0.18
Serine	118.28	13.43	10.07	10.73	7.46	10.95	0.61
Alanine	56.05	3.78	2.81	3.34	2.50	3.99	0.43
GABA	34.87	3.86	2.13	2.56	2.00	2.11	0.13
Caffeine	2261.25	305.30	332.63	373.70	301.85	402.61	269.08
(mM)	11.64	1.57	1.71	1.92	1.55	2.07	1.39
EGCG	1117.07	310.06	380.41	475.83	273.85	374.39	44.08
(mM)	2.44	0.68	0.83	1.04	0.60	0.82	0.10
EGC	477.78	211.44	303.24	357.23	292.03	180.70	32.63
ECG	232.57	68.47	87.36	104.93	60.27	78.68	17.78
EC	193.37	67.98	80.18	96.40	71.51	61.15	11.26
(+) C	28.33	8.66	9.68	13.21	7.75	9.75	22.27
GC	53.05	20.10	34.73	41.05	27.59	22.50	76.35
CG	55.36	19.28	21.18	32.94	14.70	17.34	9.83
CE/TA ratio	0.70	1.26	1.67	1.73	2.31	1.62	49.14

Values of theanine, arginine, caffeine, and EGCG converted to moles (mM) and CE/TA ratio are shown in red.

## Data Availability

The original contributions presented in the study are included in the article, further inquiries can be directed to the corresponding author.

## References

[1] Sakato Y. (1958). Chemistry of Tea. Tea Res. J. (Chagyo Kenkyu Hokoku).

[2] Nakagawa M. (1973). Relation of the Constituents with Taste of Green Tea. Tea Res. J. (Chagyo Kenkyu Hokoku).

[3] Ikegaya K., Muramatsu K. (1991). Chemistry of Tea. The Science of Tea.

[4] Research Group of Green Tea Brewing (1973). Brewing Condition of Tasty Cup of Green Tea. Tea Res. J. (Chagyo Kenkyu Hokoku).

[5] Shimotoku T., Ichikawa H., Anan T., Takayanagi H., Ikegaya K. (1982). Relation between amounts of some ingredients extracted from green tea and brewing conditions. Tea Res. J. (Chagyo Kenkyu Hokoku).

[6] Oyaizu T. (2004). Policies for the Spread of Tea Cultivars on Shizuoka Prefecture. Proc. Vege. Tea Sci..

[7] Meyer B.R., White H.M., McCormack J.D., Niemeyer E.D. (2023). Catechin Composition, Phenolic Content, and Antioxidant Properties of Commercially-Available Bagged, Gunpowder, and Matcha Green Teas. Plant Foods Hum Nutr..

[8] Unno K., Hara A., Nakagawa A., Iguchi K., Ohshio M., Morita A., Nakamura Y. (2016). Anti-stress effects of drinking green tea with lowered caffeine and enriched theanine, epigallocatechin and arginine on psychosocial stress induced adrenal hypertrophy in mice. Phytomedicine.

[9] Unno K., Furushima D., Hamamoto S., Iguchi K., Yamada H., Morita A., Horie H., Nakamura Y. (2018). Stress-Reducing Function of Matcha Green Tea in Animal Experiments and Clinical Trials. Nutrients.

[10] Unno K., Nakamura Y. (2021). Green Tea Suppresses Brain Aging. Molecules.

[11] Unno K., Taguchi K., Matsuda T., Nakamura Y. (2024). Stress-Relieving Effects of Green Tea Depend on the Ratio of Its Special Ingredients and the Infusion Conditions. Molecules.

[12] Konishi S., Muramatsu K. (1991). Biochemistry of Tea Tree. The Science of Tea.

[13] Sano T., Horie H., Matsunaga A., Hirono Y. (2018). Effect of shading intensity on morphological and color traits and on chemical components of new tea (*Camellia sinensis* L.) shoots under direct covering cultivation. J. Sci. Food Agric..

[14] Maeda-Yamamoto M., Muramatsu K., Oguni I., Isemura M., Sugiyama K., Maeda-Yamamoto M. (2002). Types of Tea and their Production Methods. Health Science of Tea, New Possibility for Physiological Function.

[15] Nakamura Y., Muramatsu K. (1991). Tea Cultivars and Cultivation. The Science of Tea.

[16] Lee L.S., Kim S.H., Kim Y.B., Kim Y.C. (2014). Quantitative analysis of major constituents in green tea with different plucking periods and their antioxidant activity. Molecules.

[17] Iwasa K., Muramatsu K. (1991). Tea Processing Science. The Science of Tea.

[18] Hirono H., Yamashita S., Hirono Y. (2024). Influence of steaming duration, chlorophyll-a and -b content and ratio, and pH on the color of green tea processed from multiple tea (*Camellia sinensis* L.) cultivars. J. Sci. Food Agric..

[19] Takayanagi H., Anan T., Ikegaya K. (1987). Changes in Physical and Chemical Characteristics of Green Tea by Different Steaming Times. Tea Res. J. (Chagyo Kenkyu Hokoku).

[20] Goto T., Horie H., Mukai T. (1993). Analysis of major amino acids in green tea by high-performance liquid chromatography coupled with OPA precolumn derivatization. Tea Res. J. (Chagyo Kenkyu Hokoku).

[21] Yamashita H., Uchida T., Tanaka Y., Katai H., Nagano A.J., Morita A., Ikka T. (2020). Genomic predictions and genome-wide association studies based on RAD-seq of quality-related metabolites for the genomics-assisted breeding of tea plants. Sci. Rep..

[22] Taniguchi T., Gejima Y., Matsuo H., Fujita S., Tatsuno T. (2012). Elution Characteristics of Catechins and Caffeine in Kamairi-cha. Tea Res. J. (Chagyo Kenkyu Hokoku).

[23] Ikeda S., Nakagawa M., Iwasa K. (1972). Relation between Infusing condition of Green Tea and Soluble Component. Tea Res. J. (Chagyo Kenkyu Hokoku).

[24] Horie H., Ujihara T., Kohata K. (2001). Elution of Major Tea Components in Tea Infusion. Tea Res. J. (Chagyo Kenkyu Hokoku).

[25] Nomura S., Monobe M., Ema K., Yamashita S., Yoshida K., Nesumi A. (2023). Differences in the Chemical Components of Cold Infusions between Sencha and Pan Fired Tea Made from the Second Crop of “Yabukita” Leaves. Tea Res. J. (Chagyo Kenkyu Hokoku).

[26] Unno K., Noda S., Kawasaki Y., Yamada H., Morita A., Iguchi K., Nakamura Y. (2017). Reduced Stress and Improved Sleep Quality Caused by Green Tea Are Associated with a Reduced Caffeine Content. Nutrients.

[27] Nakagawa M. (1970). Correlation of the Chemical Constituents with the Organoleptic Evaluation of Green Tea Liquors. Tea Res. J. (Chagyo Kenkyu Hokoku).

[28] Wada K., Nakada N., Ota I., Honjo Y. (1981). Regional Differences in the Qualities and the Chemical Compositions of Sen-cha (Common Green Tea). Tea Res. J. (Chagyo Kenkyu Hokoku).

[29] Goto T., Horie H., Ozeki Y., Masuda H., Warashina J. (1994). Chemical Composition of Japanese Green Teas on Market. Tea Res. J. (Chagyo Kenkyu Hokoku).

[30] Matsunaga A., Saba T., Nesumi A. (2009). Effect of nitrogen fertilizers on composition of catechins in shoots of tea cultivars. Tea Res. J. (Chagyo Kenkyu Hokoku).

[31] Kimura Y., Kanda M. (2013). Characteristics of the Light Spectrum Environment of *Honzu* (orthodox shading) Covering Culture and Influence of UV Irradiation or Shielding on the Quality of Tea New Shoots. Tea Res. J. (Chagyo Kenkyu Hokoku).

[32] Horie H., Ujihara T., Kohata K. (2002). Umami Taste of “Gyokuro” High Grade Green Tea. Tea Res. J. (Chagyo Kenkyu Hokoku).

[33] Mizukami Y. (2020). Identification of Key Odorants Isolated from the Headspace above Gyokuro bu Using Solid-Phase Microextraction. Tea Res. J. (Chagyo Kenkyu Hokoku).

[34] Türközü D., Şanlier N. (2017). L-theanine, unique amino acid of tea, and its metabolism, health effects, and safety. Crit. Rev. Food Sci. Nutr..

[35] Shao J., Wei Y., Wei X. (2022). A comprehensive review on bioavailability, safety and antidepressant potential of natural bioactive components from tea. Food Res. Int..

[36] Wikoff D., Welsh B.T., Henderson R., Brorby G.P., Britt J., Myers E., Goldberger J., Lieberman H.R., O’Brien C., Peck J. (2017). Systematic review of the potential adverse effects of caffeine consumption in healthy adults, pregnant women, adolescents, and children. Food Chem. Toxicol..

[37] Monobe M., Ema K., Tokuda Y., Maeda-Yamamoto M. (2010). Enhancement of the Phagocytic Activity of Macrophage-Like Cells with a Crude Polysaccharide Derived from Green Tea (*Camellia sinensis)* Extract. Biosci. Biotechnol. Biochem..

[38] Okazaki K. (2016). Microbiological Contamination of the Tea Extract Prepared with Cold Water. Tea Res. J. (Chagyo Kenkyu Hokoku).

[39] Fukushiyama E., Tokuda K., Takyu T., Saba T. (1999). Effect of Soaking Time of a Green Tea Bag in Cold Water on the Flavor and Amount of Constituents Extracted. J. Home Econ. Jpn..

[40] Hursel R., Westerterp-Plantenga M.S. (2013). Catechin- and caffeine-rich teas for control of body weight in humans. Am. J. Clin. Nutr..

[41] Jung E.S., Park J.I., Park H., Holzapfel W., Hwang J.S., Lee C.H. (2019). Seven-day Green Tea Supplementation Revamps Gut Microbiome and Caecum/Skin Metabolome in Mice from Stress. Sci. Rep..

[42] Chen S.Q., Wang Z.S., Ma Y.X., Zhang W., Lu J.L., Liang Y.R., Zheng X.Q. (2018). Neuroprotective Effects and Mechanisms of Tea Bioactive Components in Neurodegenerative Diseases. Molecules.

[43] Mancini E., Beglinger C., Drewe J., Zanchi D., Lang U.E., Borgwardt S. (2017). Green tea effects on cognition, mood and human brain function: A systematic review. Phytomedicine.

[44] Xu Y.Q., Ji W.B., Yu P., Chen J.X., Wang F., Yin J.F. (2018). Effect of extraction methods on the chemical components and taste quality of green tea extract. Food Chem..

[45] Cabrera M., Taher F., Llantada A., Do Q., Sapp T., Sommerhalter M. (2021). Effect of Water Hardness on Catechin and Caffeine Content in Green Tea Infusions. Molecules.

[46] Pérez-Burillo S., Giménez R., Rufián-Henares J.A., Pastoriza S. (2018). Effect of brewing time and temperature on antioxidant capacity and phenols of white tea: Relationship with sensory properties. Food Chem..

